# Brazilian consensus recommendations for the diagnosis, screening, and treatment of individuals with fabry disease: Committee for Rare Diseases - Brazilian Society of Nephrology/2021

**DOI:** 10.1590/2175-8239-JBN-2021-0208

**Published:** 2022-02-23

**Authors:** Cassiano Augusto Braga Silva, Luis Gustavo Modelli de Andrade, Maria Helena Vaisbich, Fellype de Carvalho Barreto

**Affiliations:** 1 Clínica de Nefrologia Senhor do Bonfim, Feira de Santana, BA, Brasil.; 2 Universidade Estadual Paulista, Botucatu, SP, Brasil.; 3 Universidade de São Paulo, São Paulo, SP, Brasil.; 4 Universidade Federal do Paraná, Curitiba, PR, Brasil.

**Keywords:** Fabry Disease, Consensus, Rare Diseases, Doença de Fabry, Consenso, Doenças Raras

## Abstract

Fabry disease (FD) is an X-linked inherited disorder caused by mutations in the GLA gene encoding enzyme alpha-galactosidase A (α-Gal A). The purpose of this study was to produce a consensus statement to standardize the recommendations concerning kidney involvement in FD and provide advice on the diagnosis, screening, and treatment of adult and pediatric patients. This consensus document was organized from an initiative led by the Committee for Rare Diseases (Comdora) of the Brazilian Society of Nephrology (SBN). The review considered randomized clinical trials, real-world data studies, and the expertise of its authors. The purpose of this consensus statement is to help manage patient and physician expectations concerning the outcomes of treatment. Our recommendations must be interpreted within the context of available evidence. The decisions pertaining to each individual case must be made with the involvement of patients and their families and take into account not only the potential cost of treatment, but also concurrent conditions and personal preferences. The Comdora intends to update these recommendations regularly so as to reflect recent literature evidence, real-world data, and appreciate the professional experience of those involved. This consensus document establishes clear criteria for the diagnosis of FD and for when to start or stop specific therapies or adjuvant measures, to thus advise the medical community and standardize clinical practice.

## Resumo

A doença de Fabry (DF) é uma doença genética, com herança ligada ao cromossomo X, que ocorre devido a variantes no gene *GLA* que codifica a enzima α-galactosidase A (α-GAL). O propósito do presente estudo foi criar um consenso objetivando padronizar as recomendações em relação ao acometimento renal da DF com orientações sobre o diagnóstico, rastreamento e tratamento de pacientes adultos e pediátricos. Esse consenso é uma iniciativa do Comitê de Doenças Raras (Comdora) da Sociedade Brasileira de Nefrologia (SBN). Foram considerados para esta revisão estudos clínicos controlados randomizados e estudos com dados de vida real somado à experiência dos autores. O resultado desse consenso foi auxiliar no gerenciamento das expectativas de pacientes e médicos quanto aos resultados do tratamento. Nossas recomendações devem ser interpretadas no contexto das evidências e ressaltando que as decisões finais devem ser tomadas individualmente, em uma decisão conjunta com o paciente e familiares, considerando os custos envolvidos, não apenas financeiros, doenças concomitantes e preferências pessoais. O Comdora pretende atualizar essas recomendações regularmente, e assim seguir novas evidências na literatura, considerar dados de vida real e valorizar a experiência profissional dos envolvidos. Esse consenso estabelece critérios claros para o diagnóstico da DF, início e interrupção de terapia específica e de medidas adjuntas, orientando a comunidade médica e uniformizando condutas.

## Definition and general aspects concerning Fabry disease

Fabry disease (FD) is an X-linked inherited disorder caused by mutations in the GLA gene encoding enzyme alpha-galactosidase A (α-Gal A). Reduced or absent enzyme activity results in gradual intralysosomal accumulation of glycosphingolipids, mainly globotriaosylceramide (GL3 or Gb3) and its metabolite globotriaosylsphingosine (lyso-Gb3)^1,2^. These deposits trigger a cascade of events, leading to alterations in energy metabolism, increased levels of inflammatory cytokines, small vessel injury, oxidative stress, and tissue ischemia, which culminate with cell dysfunction and cell death. FD affects more significantly the kidneys, the heart, and the central nervous system (CNS)^1,2^.

The GLA gene is located in the long arm of chromosome X, on position Xq22.1. More than a thousand variants have been described, some of which are benign polymorphisms without clinical significance^2,3^. Each variant tends to be family-specific and translate into variations in enzyme activity and interfamilial phenotype differences^2^. It should be pointed out that phenotype variation is observed even among patient with the same variant. Factors probably affecting the effects of a variant include the presence of additional deleterious variants or variants of unknown significance (VUS) in the GLA gene, variants in modifier genes, concurrent conditions, and environmental modifiers^2,4,5^.

Prevalence of the disease has been estimated at approximately 1:40,000 male individuals^2^. Neonatal FD screening studies have reported higher prevalence, but many variants are benign or VUS^6,7^. In populations at risk, prevalence has been estimated at 0.21% for males and 0.15% for females on hemodialysis (HD); 0.94% for males and 0.90% for females with heart disease; and 0.13% for males and 0.14% for females with stroke^8^.

Two clinical presentations of the disease have been described with variations between sexes: type 1 classic phenotype and type 2, non-classic, or late-onset phenotype.

1.a. Classic phenotype in males

Males with the classic variant present with the following characteristic findings:

- Acroparesthesias by GL3 deposition in the small fibers of peripheral nerves, principally in distal extremities; and Fabry crises, with bouts of high intensity, incapacitating pain, starting in the hands and feet and lasting from minutes to weeks. These symptoms usually start before the age of 18 years;
- Gastrointestinal symptoms such as vomiting, diarrhea, and abdominal pain after meals;
- Angiokeratomas: clustered dark red non-itchy papules occurring predominantly between the umbilicus and the knees in a swimsuit pattern, although they may also appear in the lips, umbilicus, genitals, and lower back;
- Hypohidrosis or anhidrosis secondary to sweat gland involvement leading to intolerance to temperature changes;
- Diminished hearing;
- Cornea verticillata caused by the deposition of GL3 in the cornea seen in eye examination with a slit lamp, after ruling out the use of medication such as amiodarone or chloroquine^2,9^.
- Episodes of fever generally precipitated by physical exercise, fatigue, stress, and rapid changes in temperature^2,9^.

Pain is one of the main early clinical manifestations. It affects the wellbeing and ability to perform of activities of daily living of patients and is seen in 60% to 80% of boys with the disease. Episodes of pain start typically at the ages of 3-10 years in boys and later in girls^10,11^.

As age increases and GL3 deposits in target organs accumulate, in the fourth decade of life patients may develop acute myocardial infarction (AMI), heart failure (HF), stroke, and chronic kidney disease (CKD). The summation of these factors decreases mean life expectancy by 20 years in males and 15 years in females^2,9^.

- Kidney involvement in FD is multifactorial and characterized by an unclear pathogenesis. GL3 deposition occurs in all kidney cells, leading to hypertrophy of endothelial cell and podocytes in particular, resulting in cell injury, podocyturia, and podocyte foot process effacement^12^. Other findings include smooth muscle cell proliferation, release of inflammatory and profibrotic mediators, increased oxidative stress, vascular lumen obliteration, and ischemia^13-15^, leading to progressive glomerulosclerosis, capillary wall thickening, tubular atrophy, interstitial fibrosis, and arterial and arteriolar sclerosis^16-19^. Glomerular manifestations are similar to the ones seen in diabetic nephropathy, with hyperfiltration in the early stages, albuminuria, proteinuria, and gradual decrease of the glomerular filtration rate (GFR)^20,21^; as a result, untreated males with classic phenotype disease in particular may develop end stage renal disease (ESRD) between the fourth and fifth decade of life^19,22^.
- Heart involvement: about 50% of the patients present with left ventricular hypertrophy (LVH), arrhythmia, angina, and dyspnea. Arrhythmia and bradycardia stem from the involvement of the sinus node, the conduction system, and sympathetic/parasympathetic system imbalance^2^. Diastolic dysfunction and concentric LVH often occur in the fourth decade of life^23^. Myocardial fibrosis sets in gradually and preferentially affects the posterolateral wall of the heart^24^. Malignant arrhythmias may be fatal^25^.
- CNS involvement manifests through a wide array of events, including headaches, vertigo and dizziness, transient ischemic attack (TIA), and ischemic stroke. The incidence of stroke is higher among patients with FD when compared with the general population paired for age^26^.

1.b. Non-classic, or late onset phenotype in males

Males with variants related to the non-classic phenotype do not present or develop milder forms of the characteristic manifestations associated with FD^27^. Cardiac involvement in FD occurs more commonly as concentric LVH around the fifth decade of life, with dilated cardiomyopathy, hypertrophic obstructive cardiomyopathy, and idiopathic cardiomegaly as conditions primarily listed in differential diagnosis^2^. Kidney involvement in FD presents signs typically seen in other forms of renal impairment along with gradual decline of the GFR, which becomes more evident around the age of 50 and develops into ESRD^2,9^.

1.c. Phenotype in females

In females, the phenotype is heterogeneous due to the random inactivation of the X chromosomes (XCi)^28^. Enzyme activity varies and may be normal. Thus, the diagnosis of FD in females must be based on the identification of the genetic mutation associated with the disease. In terms of clinical signs, female patients have been described as presenting no symptoms to developing classic phenotype FD similarly to males^2,29^.

## Goals of the Brazilian Consensus for Fabry Disease (Comdora-SBN)

This Consensus document was developed in an initiative coordinated by the Committee for Rare Diseases (Comdora) of the Brazilian Society of Nephrology (SBN) to standardize recommendations related to kidney involvement in FD in the areas of diagnosis, screening, and treatment of adult and pediatric patients.

Although consensus documents have been published in other nations, it is important to develop national consensus documents to summarize evidence while taking into account regional experience and country specificities. Vast experience has been amassed in Brazil about the diagnosis, management, and treatment of patients with FD. This consensus document considers existing evidence along with national specificities.

## Methods employed in the production of recommendations

A panel of Brazilian experts was convened to develop a consensus document on the diagnosis and treatment of FD based on their respective personal experiences and a literature review. A narrative review of the literature ensued from searches performed on databases Medline, PubMed, and Cochrane Library with keywords “Fabry” and “Fabry disease” without language restrictions and including papers published until June 2021.

Organizing a randomized controlled trial about Fabry disease is inherently difficult, since this is a condition with very few cases reported. Based on recommendations from the literature on rare diseases, we included methodologically less rigorous studies describing real-world data in our review. Case series, cohort studies, and registry studies were thus considered^30^. Additionally, the experience of the authors, particularly in controversial points, was taken into account.

The themes that guided the production of this consensus document were:

1. Definitive diagnostic criteria for FD;
2. Screening indications and recommendations;
3. Treatment indications;
4. Treatment discontinuation indications;
5. Differences between available therapies;
6. Kidney involvement and progression to Fabry nephropathy.

The Comdora group conducted the literature review and the meetings of the panel of Brazilian experts. This paper presents the consensus reached by specialized working groups tasked with the development of therapeutic goals focused on kidney involvement and the agreement over the goals for the treatment of other systemic manifestations stemmed from FD.

The evidence and recommendation classes alluded to throughout the text are described in Table 1. They are Class I (recommended), Class II (potentially recommended), and Class III (not recommended)^31^. The quality of evidence was judged based on clinical experience, observational studies, available randomized studies, and previously published guidelines.

**Table 1:** Evidence/recommendation classes

Class I	Evidence and/or general agreement that a given treatment or procedure is beneficial, useful, effective.	Is recommended/indicated.
Class II	Conflicting evidence and/or divergence of opinion about the usefulness/efficacy of the given treatment or procedure.	
Class IIA	Weight of evidence/opinion is in favor of usefulness/efficacy.	Should be considered.
Class IIB	Usefulness/efficacy is less established by evidence/opinion.	May be considered.
Class III	Evidence or general agreement that the given treatment or procedure is not useful/effective, and in some cases may be harmful.	Is not recommended

A total of 127 references were included in the review, most of which observational studies (n = 50; 39%). Randomized studies accounted for 8.6% (n = 11) and registry studies for 5.5% (n = 7) of the references; this is a common split for rare diseases, for which observational studies are an important source of evidence. Other consensus documents on FD were included in the review (n = 12; 9.4%), along with experimental studies (n = 5; 3.9%), websites (n = 3; 2.3%), and other sources (n = 2; 1.5%).

The points in which author experience contributed more significantly with the conclusions were definitive diagnosis, screening indications, and comparisons between the two enzyme replacement therapies available.

## Clinical suspicion

Individuals showing previously described signs and symptoms and a family history of the condition should be suspected for Fabry disease.

## Diagnostic confirmation

### Measurement of enzyme activity

The first step to confirming a diagnosis of FD is to measure the activity of enzyme α-GAL, which can be done via plasma, white blood cells, or through the dried blood spot (DBS) method. Males with classic variants have very low (< 5%) or absent enzyme activity levels, while late-onset cases present variable enzyme activity levels (5-30%). Although this is a highly sensitive method for males, its specificity is compromised by issues with sample transportation and integrity, which may yield false results when activity levels are low^32^; in such cases, patients must be retested with a different test type (plasma or white blood cells). Symptomatic females with FD may present normal to slightly diminished enzyme activity levels, and such finding does not rule out a diagnosis of FD^2,33^.

### Gl3 and lyso-gl3

Plasma and urinary GL3 and lyso-GL3 are biomarkers of FD. Although GL3 levels are commonly elevated in patients with the disease - and thus serve as a good indication of response to specific therapy, a linear correlation does not necessarily exist between biomarkers and clinical manifestations^34,35^. Normal biomarker levels do not rule out a diagnosis of FD, particularly for females^36^.

Plasma lyso-GL3 is more sensitive and specific for patients of either of the sexes, correlates with FD phenotype, and may be elevated in females with normal enzyme activity^37,38^. It may serve as a predictor of pathogenicity and supports diagnosis in cases of new genetic variants, VUS, or in the absence of variants^32,39,40^. It inhibits α-GAL activity and plays an important role in FD nephropathy by causing smooth muscle cell proliferation and the release of glomerular injury mediators^41-43^.

### Genetic testing

The detection of the causing mutation confirms diagnosis of the disease. Genetic testing is of paramount importance in the definitive diagnosis of female patients and may direct treatment and the screening of family members of male and female patients^44^. In the presence of a probably or definitely pathogenic mutation, diagnosis is reached without doubt; however, when VUS or new variants are detected, diagnostic confirmation requires the investigation of its associations with phenotype and biomarkers and even the use of in silico prediction tools. In this consensus document, we recommend that doubtful cases be assessed by a specialist on FD with the support of a geneticist, if needed.

### Test interpretation and the diagnostic challenges of FD

Females suspected for FD may benefit from a combined biochemical and genetic approach. The measurement of enzyme activity combined with lyso-GL3 levels substantially improves diagnostic accuracy. Abnormal levels in both tests have yielded a positive predictive value (PPV) of 97% in the confirmation of cases of FD. When only one of the tests presents altered results, elevated lyso-GL3 has been described as a more sensitive indicator than diminished enzyme activity, with a PPV of 39% vs. 6%, respectively. Therefore, approximately 60% of the females with FD would not be diagnosed if enzyme activity were used in isolation^(45)^. The ratio between α-GAL and lyso-GL3 in females was 100% sensitive at distinguishing between individuals with the disease and controls, and is thus a useful screening tool for female subjects^46^.

The diagnosis of FD is challenging when patients do not present with typical symptoms, as in cases detected from family screening and in females, in which the severity of involvement depends on the variant and on the pattern of XCi^47^. Note that genetic analysis may be inconclusive and reveal potentially benign variants or VUS. Additionally, a variant previously described as a VUS may have its pathogenicity confirmed or vice-versa. Queries in mutation databases are needed in order to verify the pathogenicity of a mutation^48,49^. Definitive diagnosis must be based on the association of phenotype and complementary workup (including genetic tests)^50^.

Table 2 lists standardized diagnostic criteria for FD for each of the sexes based on previously published protocols^51,52^. Diagnosis is based on the presence of a genetic mutation combined with clinical, biochemical, and histology findings, and patient family history of disease.

**Table 2:** Criteria for the definitive diagnosis of FD

Males		Females	
Presence of genetic mutation		Presence of genetic mutation	
+		+	
a-GAL deficiency ≤ 5%		Measurement of a-GAL not needed	
+			
A or B or C or D #			
A (clinical)	B (biochemical)	C (familial)	D (histology)
Presence of one of more of the following: neuropathic pain, cornea verticillata, angiokeratoma	Elevated plasma or urinary GL3 or lyso-GL3 (> 1.8ng/mL)	Family member with definitive diagnosis of FD carrying the same mutation	Histology alterations suggestive of lysosomal deposits in target organs (kidneys, skin, heart)

Legend: FD (Fabry disease); α-GAL (α-galactosidase A); GL3 (globotriaosylceramide); lyso-GL3 (globotriaosylsphingosine).

# Exception: males with pathogenic mutation (class I) and α-GAL activity ≤ 5%, not meeting other criteria (A/B/C/D).

As shown in Table 2, the diagnosis of FD in males requires the presence of a mutation associated with disease and decreased enzyme activity (< 5%), with or without clinical (A), biochemical (B), family (C) or histology (D) criteria^53^. In females, the measurement of enzyme activity may be unnecessary, since it may be normal^54^, whereas criteria A or B or C or D must be present. The family criterion includes the presence of a relative with FD with the same genetic mutation, while histology includes the detection of GL3 tissue deposits. In terms of genetic testing, doubt comes up with the detection of a VUS, similarly to the case of new variants in patients with LVH, early stroke or proteinuria who do not meet the criteria for a definitive diagnosis of FD. In these cases, the gold standard diagnostic finding is the detection of GL3 deposits in kidney or heart biopsy specimens, with the aid of electron microscopy^22^. In these cases, therefore, the histology criterion prevails.

Similarly to previously published expert consensus documents, the algorithm in Table 2 tries to correlate genotypes and phenotypes^54-56^, allowing the assignment of patients to classic or non-classic FD categories.

Non-classic disease is generally characterized by the presence of a genetic mutation and involvement of a specific organ, without the other criteria for classic FD. Since phenotypes vary even among patients with the same variant, plasma lyso-GL3 may contribute with disease categorization. Lyso-GL3 levels are similar in males with non-classic FD and females with classic FD^57^.

## Screening recommendations

### Screening of family members from an index case

Systematic screening of family members of individuals with FD is a simple and effective way to attain early diagnosis. After an index case has been found, building a pedigree covering at least three generations and investigating every family member - even asymptomatic ones - for X-linked inheritance pattern is recommended. On average, five family members are diagnosed with FD for each index case, with some studies featuring even greater numbers^58,59^. Detailed clinical history coupled with physical examination may find subjects with incipient disease.

**Figure 1:**
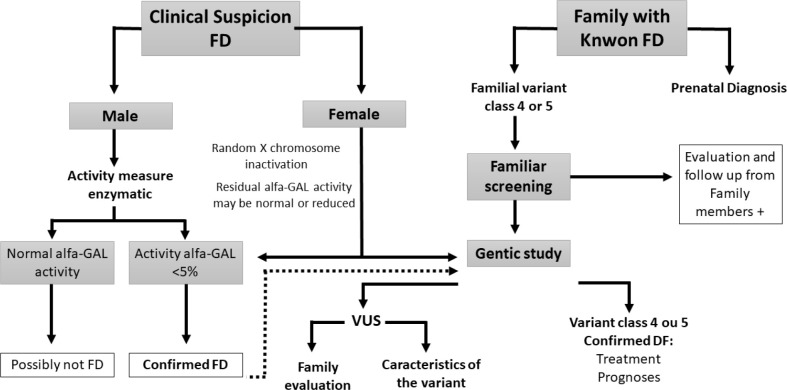
Fabry disease diagnostic identification and investigation flowchart.

The first step in screening is conducting thorough interviews to capture the family history of disease and select individuals suspected for FD^60^. Next is measurement of enzyme activity in males; if results are 25-30% below the average levels seen in controls, genetic testing is warranted^56^. Females should undergo genetic testing right away. Plasma lyso-GL3 plays an important role in doubtful cases. Figure 1 shows a workflow recommended for the investigation of an index case and other cases detected from family screening.

### Screening of populations at risk

Screening for FD is recommended for patients categorized as belonging to populations at risk of disease, a group that includes subjects with kidney disorders such as proteinuria or albuminuria, individuals with stage 5D CKD, heart disease such as hypertrophic cardiomyopathy, or cerebrovascular disease such as stroke or TIA not explained by other causes. Screening helps to identify an index case and diagnose other affected family members.

Individuals with clinical signs indicative of FD should be investigated regardless of preexisting cases of the disease in their families, since phenotype variability is substantial and “de novo” variants may occur.

Differently from other protocols, in our region of the world we investigate males aged 50+ years^60^, since access to the health care system is often precarious and knowledge of the underlying disease by populations at risk is minimal, with many patients being diagnosed with late-stage disease and severe symptoms^61^. In support of this recommendation, studies have reported the detection of classic FD in males on renal replacement therapy aged 50+ years^59^. Besides, it is important to realize that the etiology of hypertensive nephrosclerosis and chronic glomerulonephritis described in diagnostic reports is mostly categorized as unknown^62^. For this reason, patients diagnosed with these conditions should not be excluded from FD screening efforts. Additionally, we must consider the possibility of FD coexisting with other causes of CKD. Therefore, if suspected for FD, patients with conditions known to cause CKD should also be investigated for FD. Since females may present with late manifestations of the disease, screening is recommended, regardless of age, for subjects with CKD, hypertrophic cardiomyopathy, or cerebrovascular disease of unknown etiology.

### Neonatal screening

Prevalence of FD reported in neonatal screening programs has been higher than in previous studies. However, there is doubt about the actual benefits of reporting higher prevalence numbers^6,7^, since many of the found genetic variants are benign or polymorphisms. Other issues include the psychological and social conflicts affecting families as they find they may have the disease, along with ethical, legal, and financial implications that may surface from the detection of a late-onset variant. On the other hand, early detection may improve prognosis and allow timely monitoring and therapy initiation to mitigate or prevent long term complications^63,64^. This consensus document does not support the instatement of systematic screening for FD in the general population. However, this position may be reviewed in light of novel knowledge and therapies.

Screening indications are summed up in Table 3.

**Table 3:** Screening indications for FD

Screening of the general population is not recommended at the moment.
We recommend screening families from an index case.*
We recommend obtaining patient consent using a properly designed form before screening.
We recommend screening individuals of all ages with kidney, heart, or neurological disorders or clinical signs or symptoms suggestive of FD without a defined etiology.
We recommend screening females of all ages with kidney, heart, or neurological alterations of undefined etiology or with symptoms potentially attributable to FD.
We recommend discussing the implications of a diagnosis of FD with the patient and involve a specialist on FD if questions about the genetic tests are asked.

Legend: FD (Fabry disease).

## Clinical management of adult patients with FD

The management of patients with FD must observe the following steps:

1. Establish a diagnosis of FD in accordance with the criteria set out in Table 2.
2. Check for target organ involvement and indication of specific therapy initiation considering the level of evidence, which is higher for cardiac or kidney indications. Males with classic variants may be treated before the onset of clinical or histology manifestations, preferentially based on the opinion of a panel of experts^47^.
3. Assess whether contraindications for therapy initiation exist.
4. Define therapeutic targets and develop a monitoring plan.
5. Follow disease progress and review response to therapy. If therapy fails to achieve the stipulated goals or if new situations arise, check the criteria to change or discontinue therapy^36^.

The management of FD is founded on the adoption of an individualized approach, which considers the natural history of each genetic variant, the early initiation of specific therapy when indicated, the use of adjuvant evidence-based measures, the monitoring of organ involvement in asymptomatic and treated patients, individuals with non-classic disease, and females, and a multidisciplinary approach throughout the stages of the disease.

### Specific therapy for FD

Before specific therapies were available, patients were given palliative care to manage symptoms^9^. Specific treatment, initially with enzyme replacement therapy (ERT) and more recently with pharmacological chaperones, also focuses on the reversion of the alterations caused by FD, on preventing disease in young patients, and on mitigating organ involvement progression. ERT has changed the lives of patients by improving pain management and ameliorating cardiac and kidney parameters, increasing survival, and improving patient quality of life^65,66^. The decision of the physician responsible for prescribing therapy must be based on the high probability of providing clinical benefit associated with the low risk of producing adverse events.

### Enzyme replacement therapy

The clinical use of ERT was approved in Europe in 2001 and in the United States in 2003^2^. Two enzymes are currently available: agalsidase alfa (Replagal^TM^), produced from fibroblast cultures and approved for use in Europe, and agalsidase beta (Fabrazyme^TM^), obtained via recombinant DNA technology from a Chinese hamster ovarian cell expression system and approved for use in Europe and the United States^67,68^. Anvisa has approved the use of the two medications in Brazil.

Studies have suggested that progression of kidney disease is attenuated in patients started on ERT at a younger age with preserved kidney function, thus corroborating early intervention^69-71^. In adults, higher urinary protein levels have been associated with higher urinary protein levels during the follow-up of males on agalsidase alfa for ten years^72^. Higher risk of gradual GFR decreases despite ERT has been observed when the baseline urinary albumin-to-creatinine ratio (ACR) is ≥ 1,000mg/g^73^. However, patients with similar levels of urinary albumin excretion may also respond differently, depending on the level of kidney damage prior to treatment^74-76^.

A prospective study with 57 adult patients (30 males) and six adolescents found GFR decreases in males on ERT (-3.4 mL/min/1.73 m2/year), while in females the GFR decrease followed the natural course (-0.8 mL/min/1.73m2/year) of gradual GFR decrease. In this study, long term ERT combined with support measures did not prevent progression to nephropathy, although longer treatment time diminished the risk for other complications^77^.

### Which enzyme should you prescribe, alfa or beta?

There is controversy over which type of ERT to prescribe. Some say that this is a matter of dosage, and conclude that the higher doses of agalsidase beta might be more effective than the lower doses indicated for agalsidase alfa. Others say that the two molecules are not absolutely equal, and that there is a difference in composition related to glycosylation and cell uptake mediated by the mannose 6-phosphate receptor^39,40^ and that, therefore, the indicated dose of agalsidase alfa is different. Pivotal studies used by regulatory agencies for the approval of these medications have described the two drugs as effective at insert-recommended doses.

The two drugs are administered intravenously every 15 days, since the enzymes in question are rapidly depleted in plasma^2,9^. The recommended doses for agalsidase alfa and beta are 0.2 mg/kg/dose and 1 mg/kg/dose, respectively^67,68^. Differently from agalsidase alfa, agalsidase beta always requires pre-medication^67,68,78^.

### Adverse effects secondary to ert:

One of the main adverse events is infusion reaction characterized by fever, rigors, edema, skin rash, nausea, dyspnea, and development of anti-agalsidase antibodies. Anti-IgG antibodies have been associated with infusion reaction, in vitro inactivation of agalsidase, and evidence of absence of response, such as elevated levels of GL3 or lyso-GL3^79,80^. The formation of anti-IgG antibodies is relatively common and has been reported with both enzymes in males with classic variants of the disease^80-83^. However, more studies are needed to assess the impact of anti-IgG antibodies on the efficacy of ERT^38,79^.

### Comparison between agalsidase alfa and beta in studies enrolling adults

Ten-year follow-up data with serial biopsies of males with classic FD have shown that the elimination of podocyte deposits of GL3 and the reduction of plasma lyso-GL3 levels were correlated with cumulative enzyme dose^84^.

A prospective observational study in which patients on agalsidase beta were switched to agalsidase alfa for shortages of agalsidase beta and then switched back to agalsidase beta once inventories normalized found that some of the benefits of the therapy were dose-dependent, such as decreases in the GFR and lyso-GL3 levels^85^.

A retrospective multicenter cohort study with 387 patients on ERT found that decreases in plasma lyso-GL3 were more marked in males with classic phenotype FD on agalsidase beta, while the GFR remained similar in both groups^86^.

The development of anti-IgE antibodies has also been reported among patients on agalsidase beta along with an association with anaphylaxis^80-83,87^. This is an important factor, since administration of agalsidase beta requires infusion at a specialized center for reasons of safety.

### Chaperones

Chaperones are another class of specific therapy. Migalastat (galafold®) was the first chaperone approved for FD, with clinical use recently approved in Brazil^88^. This medication is indicated only to patients with amenable variants (susceptible to the drug) of the missense type. Migalastat selectively and reversibly binds to mutated forms of α-GAL, promoting enzyme stability within the endoplasmic reticulum and facilitating its transportation to the lysosomes, where the bond is undone, culminating with proper enzyme function. The drug is given orally and offers good tissue distribution. Unlike ERT, migalastat crosses the blood-brain barrier^89,90^.

The efficacy of migalastat was assessed mainly in two trials. The FACET study reported a decrease greater than 50% in interstitial inclusions in peritubular capillaries, a significant reduction in podocyte inclusions, and improved kidney function, regardless of baseline urinary protein levels, after six months of treatment^91^. In the ATTRACT randomized trial, migalastat and ERT had similar effect over kidney function 18 months into the study^92^, although migalastat increased α-GAL activity, stabilized kidney function, and kept plasma lyso-GL3 levels low in a subgroup of Japanese patients^93^. Another study also observed a decrease in podocyte deposits of GL3 after six months of treatment with migalastat^94^. Effective stabilization of the GFR and reduction of kidney deposits of GL3 were reported in males with classic phenotype FD and in other groups of patients with less severe disease. The quantity of podocyte deposits was the only item rated as stable by the end of the follow-up period in the group of patients with classic phenotype disease^95^.

Table 4 shows current therapy options and recommendations regarding dosage, indications, and contraindications.

**Table 4:** Information about specific therapy for FD

Medication	Dose/route of administration	Periodicity	Variant treatment indication	Age to start therapy as indicated in insert	Contraindications
ERT					
Agalsidase alfa	0.2 mg/kg Intravenous	15/15 days	Any*	7 years	Severe infusion reaction
Agalsidase beta	1.0 mg/kg Intravenous	15/15 days	Any *	8 years	Severe infusion reaction/presence of anti-IgE antibodies
Chaperone					
Migalastat	1 capsule (123 mg) Oral	Every other day	Amenable variants*+	16 years	GFR < 30mL/min/1.73m^2^

Legend: FD (Fabry disease), ERT (enzyme replacement therapy), GFR (glomerular filtration rate).

* Presence of variant associated with definitive diagnosis of FD.

+ Susceptible mutations in in vitro testing (HEK test).

It is important to mention that in the cases of patients with amenable variants it is up to the physician jointly with the patient to assess the favorable points of each therapy while deciding between ERT and chaperones. ERT with agalsidase alfa or beta has been approved for use in individuals aged seven or older and eight or older, respectively^67,68^, while migalastat can be prescribed to patients aged 16 years or older with a GFR greater than 30 mL/min/1.73m^2^.

### Other therapies under research

Novel treatments being developed include glucosylceramide synthase inhibitors, a drug class that decreases the production of glycosphingolipids in an approach known as substrate reduction therapy^96,97^. Lucerastat, the most widely studied compound, can be used with other therapies. However, it is still the topic of preliminary phase 1 trials^96,97^.

### Indications to start specific therapy

Below are the recommendations to start specific therapy for each case of the disease.

- **Symptomatic and asymptomatic males with classic FD:** Specific therapy is indicated at any age upon diagnosis, since it delays or prevents the progression of FD before the installation of irreversible alterations^98^; however, some authors believe that therapy should commence only when signs of organ involvement are present^47,51^.

Some data may support the indication of early therapy initiation, such as a family history of severe disease in males, inability to detect α-GAL activity, and elevated plasma lyso-GL3^99^. The decision to start treatment must be shared between the physician and patient family, considering the challenges inherent to undergoing bimonthly intravenous infusion sessions. The administration of infusions at home is a good option for patients who tolerate treatment well, and is usually recommended to subjects on agalsidase alfa with good results in terms of compliance and safety^100^. Studies have attested to the safety of agalsidase beta home infusions^101^.

Considering the above, we recommend that ERT should be offered to males with classic FD from the age of seven years, even in the absence of signs or symptoms (CLASS IIA RECOMMENDATION).

- **Males and females with classic phenotype disease** must be treated as soon as early signs of FD-related target organ involvement are present (CLASS I RECOMMENDATION)^51^.
- **Symptomatic females:** Always initiate specific treatment.
- **Asymptomatic females:** Start specific therapy if there is workup or histology evidence of kidney injury, such as a GFR of less than 90 mL/min/1.73m^2^, an ARC persistently greater than 30 mg/g, or foot process effacement, moderate or severe GL3 inclusions and signs of glomerulosclerosis in kidney tissue.
- **Adult male or female subjects with VUS** must be treated when there is biochemical or histology evidence of FD-related kidney involvement, even if other symptoms are absent (CLASSE IIB RECOMMENDATION).

### Indications directed specifically to kidney involvement

The Canadian consensus statement suggests that males with kidney disorders and/or urinary protein greater than 500 mg/24 hours or histopathology alterations require treatment^55^. Glomerular hyperfiltration (GFR > 135 mL/min/1.73m^2^) is a minor criterion to initiate therapy in Canadian guidelines^55^. The European consensus recommends that treatment for males with pathogenic variants should be initiated in the presence of albuminuria, proteinuria, or CKD stages 1 or 2 (GFR between 60 and 90 mL/min/1.73m^2^ - CLASS I RECOMMENDATION), and individuals with stage 3a CKD (GFR between 45 and 60 mL/min/1.73m^2^ - CLASS IIB RECOMMENDATION)^51^. Treatment is not contraindicated for patients on dialysis, even when they are not eligible for kidney transplantation, or in patients with cognitive decline for any cause. In these cases, assessment must be individualized^51^.

Other authors do not recommend the initiation of therapy for patients with proteinuria greater than 1 g/day or a GFR below 60 mL/min/1.73m^2^, except for non-renal indications. Thus, they recommend that therapy must be maintained for patients with advanced CKD (GFR below 45 mL/min/1.73m^2^) or kidney transplant patients, given its relevance to additional involvement derived from FD^60^.

The updated European consensus document recommends that treatment be initiated in male patients with classic phenotype upon diagnosis, even in the absence of albuminuria. Treatment for males and females with non-classic phenotypes should be initiated in the presence of albuminuria^47^.

As recommendations in this consensus document, treatment is indicated for males with urinary protein and/or proteinuria (ARC greater than 30 mg/g) and/or mild to moderate CKD (GFR greater than 60 mL/min/1.73m^2^) related to FD (CLASS I RECOMMENDATION). Treatment is not formally indicated for patients with advanced CKD (CLASS IIA RECOMMENDATION); however, therapy is indicated even to patients with CKD stages 5 or 5D or transplant patients for involvement of other organs based on individualized assessment (CLASS IIB RECOMMENDATION). Given the particularities cited above, in females the treatment recommendation classes are slightly different, as described in Table 5.

**Table 5:** Indications for when to start therapy based on kidney disorders

Definitive diagnosis of FD	
+	
Males	Females
Albuminuria*(CLASS I)	Albuminuria*(CLASS IIA)
Proteinuria* (CLASS I)	Proteinuria* (CLASS IIA)
CKD (GFR 60-90) (CLASS I)	CKD (GFR 60-90) (CLASS IIA)
CKD (GFR < 60) (CLASS IIB)	CKD (GFR < 60) (CLASS IIB)
Histology alteration# (CLASS I)	Histology alteration # (CLASS IIB)

Legend: FD (Fabry disease), CKD (chronic kidney disease), GFR (glomerular filtration rate).

* In the absence of other causes of microalbuminuria or proteinuria.

# Biopsy findings consistent with FD histology alterations.

The presence of FD-related histology alterations such as GL3 deposits in podocyte cells amount to treatment indication, even in the absence of clinical signs of kidney involvement such as proteinuria/microalbuminuria (CLASS I RECOMMENDATION).

In kidney histology, the presence of GL3 deposits, mesangial expansion, glomerulosclerosis, tubular atrophy, and interstitial fibrosis has been observed in the early stages of disease before the onset of albuminuria^2,102^. Therefore, although albuminuria/proteinuria are the most widely used markers in clinical practice, their sensitivity is low when it comes to identifying incipient nephropathy^56^. In addition, proteinuria might not be evident in patients with advanced kidney disease and may not be related to GFR decline^37^.

The recommendation is that kidney alterations should be assessed via the measurement of albuminuria and proteinuria in isolated urine samples (corrected for urinary creatinine) or 24-hour urine tests, and that the GFR be calculated using the CKD-EPI equation for adult patients or measured via 24-hour urine collection^47,103^.

Patients aged 50+ years do not have a clear-cut indication about when to initiate treatment. If analyzed in isolation, being older than 50 years is not a contraindication in itself, although studies enrolling individuals in this age range are lacking. Symptom-based indication may be beneficial and more economical than initiating therapy to prevent clinical events and progression of FD. The decision to start or continue therapy in the long term must be individualized and consider the cost-effectiveness of the intended measures^104^. It is important to realize that the presence of kidney signs and symptoms in patients aged 50+ years may simply reflect natural aging^105^.

Patients failing to meet the criteria for therapy upon diagnosis must be monitored periodically for FD-related organ involvement and have therapy initiated as soon as it becomes needed. The recommendations for the initiation of treatment for adult patients are listed in Table 6.

**Table 6:** Recommendations for when to start specific therapy in adult patients with classic mutations, late-onset disease, or VUS

Classic Variants
**Male patient, symptomatic or asymptomatic** Therapy must be considered and applies to all patients at any age of presentation.
**Female patient, symptomatic** Signs and/or symptoms suggestive of kidney involvement associated with FD: - Proteinuria/albuminuria not attributable to other causes; - Evidence of kidney dysfunction (may require kidney biopsy if isolated).
**Female patient, asymptomatic**
Therapy must be considered if workup, histology, or kidney injury imaging evidence is available, such as persistently decreased GFR (< 90 mL/min/1.73m2); ACR > 30 mg/g; kidney biopsy showing signs of foot process effacement or glomerulosclerosis accompanied by moderate to severe GL3 inclusions in different kidney cell types.
**Late-onset disease or VUS**
**Male and female patients**
- Therapy must be considered and is adequate if workup, histology, or kidney injury imaging evidence is available, even in the absence of typical symptoms of FD. Anomalous findings must be associated with FD, which might require histology testing or the assessment of biochemical evidence of GL3 accumulation. - Advice from a geneticist or a specialist in FD may help interpret the pathogenicity of a VUS. - Individuals with well-characterized benign polymorphisms should not be treated. - If tissue involvement or clinical symptoms linked to FD are absent, therapy may not be adequate, particularly for females. .

Legend: VUS (variant of unknown significance), FD (Fabry disease), GFR (glomerular filtration rate), ACR (albumin-to-creatinine ratio).

### Indications of kidney biopsy for adult patients:

- Patients with minimal proteinuria and normal kidney function should be biopsied to check for significant GL3 deposition, particularly in podocytes, which may indicate the need to start therapy^102^.
- Females without clinical evidence of FD nephropathy should be biopsied to check for significant kidney deposits and indications to initiate specific therapy^34^.
- Kidney biopsy might be needed to assess overlapping nephropathies and cases with atypical presentations for purposes of developing differential diagnosis^102,106-108^.
- Kidney biopsy might be needed to assess response to therapy (new biopsy);
- Kidney biopsy might be indicated for patients with established glomerular hyperfiltration even if without proteinuria.

Kidney biopsy might be useful in every patient with any level of proteinuria or kidney dysfunction to assess the degree of glomerulosclerosis and interstitial damage, which are markers of chronicity of great prognostic value^34^.

### Contraindications to start therapy

For some patients diagnosed with FD, there are situations in which specific therapy is not indicated. Treatment is not recommended for patients with CKD stages 4 or 5 ineligible for kidney transplantation with NYHA class IV HF or any advanced disease leading to a life expectancy of less than a year^51,55^. The presence of anti-IgE antibodies against agalsidase is generally considered an absolute contraindication given the risk of anaphylactic reaction^55^. In these cases, since the appearance of IgE is often associated with the use of agalsidase beta, there is the possibility of swapping it for agalsidase alfa. Nevertheless, some authors advocate the maintenance of agalsidase beta infusions via de-sensitization protocols^109,110^.

Treatment must be assessed individually in the cases of patients with a GFR below 45 mL/min/1.73m^2^, individuals on renal replacement therapy, and subjects with cognitive decline, considering the benefits it offers to other organs.

Pregnancy is a relative contraindication for ERT. Successful pregnancies have been reported among patients on either type of ERT^111,112^. Migalastat is contraindicated during pregnancy for lack of safety data. Females must be advised to discontinue therapy before conceiving and while they are breastfeeding, and to use contraceptives^88^.

The contraindications to start specific therapy are described in Table 7.

**Table 7:** Contraindications to start specific therapy

When NOT to indicate treatment/recommendation classes (Males and Females)
Patients with CKD ineligible to kidney transplantation with advanced HF - NYHA class IV (CLASS IIA)
Primary renal indication: Stage 5 CKD (CLASS IIA)
Advanced FD or other comorbidities leading to a life expectancy of less than a year (CLASS IIB)
Severe cognitive decline for any cause (CLASS IIB)
Other conditions in which the benefits from therapy do not pay off (CLASS III)
Anaphylactic reactions from use of ERT associated with the presence of IgE (CLASS III)

Legend: CKD (chronic kidney disease); HF (heart failure); NYHA (New York Heart Association); FD (Fabry disease); ERT (enzyme replacement therapy).

### Indications to suspend therapy

Poor compliance (patients missing more than 50% of infusion sessions), patients lost during follow-up, and patients unwilling to be treated rank among the top indications to suspend therapy. Patients meeting contraindication criteria (Table 7) during treatment must be assessed for therapy discontinuation^52,56^. In this consensus document, the presence of severe reactions to ERT was deemed as an indication to discontinue therapy (CLASS I RECOMMENDATION) or change medication.

The criteria to suspend therapy apply to patients of all sexes with classic or non-classic FD. However, if the indication for ERT derives from neuropathic pain, lack of response is not an indication to discontinue therapy for males with classic FD, since these patients are at high risk of vital organ involvement^51^.

### Adjuvant therapies

Specific treatment for FD must be combined with support measures directed to target-organ and chronic tissue injury complications. Preventive measures and lifestyle modifications must be considered in the overall management of patients^2^.

In cases of FD nephropathy, the guidelines for the treatment of CKD must be followed, including measures to control systemic hypertension and promote smoking cessation, along with individualized diets and dyslipidemia therapy.

Renin-angiotensin-aldosterone system (RAAS) blockade using angiotensin-converting-enzyme (ACE) inhibitors or angiotensin II receptor blockers (ARBs) is an important measure, since these drugs decrease proteinuria and offer cardioprotection^34^. Table 8 describes therapeutic goals. Blood pressure (BP) targets are as follows: Systolic BP ≤ 130 mmHg and diastolic BP ≤ 80 mmHg^113^. Dose must be titrated to prevent adverse events such as hypotension and hyperkalemia^47,76,114^. Patients must be monitored for kidney function and doses adjusted or medication discontinued if the GFR declines. Age at the start of ERT might interfere with proteinuria and GFR preservation goals^75,76^.

**Table 8:** Therapeutic goals for kidney manifestations of FD

GFR (mL/min/1.73m2)	
No kidney involvement	Avoid or mitigate GFR decline
Mild kidney involvement: Normal GFR (90-120) or hyperfiltration (> 120)	Keep the GFR within the normal range for the patient’s age.
Mild to moderate involvement (GFR 60-90)	Stabilize or mitigate GFR decline.
Moderate to severe involvement (GFR 30-59)	Avoid progression of GFR decline to delay or prevent CKD stage 5 or 5D.
Severely decreased GFR (15-29)	Decrease the GFR decline as much as possible. Delay progression to CKD stage 5 or 5D.
CKD stage 5 or 5D	Provide ideal RRT (dialysis or kidney transplantation). Keep ERT to avoid damage to the heart and CNS. Encourage preemptive transplantation.
**Albuminuria (mg/g)**	
General: All patients	Keep albuminuria at the lowest level possible.
Urinary albumin excretion: 30-300	Normalize or stabilize urinary albumin excretion.
Urinary albumin excretion: > 300	Decrease urinary albumin excretion to < 300.

Legend: GFR (glomerular filtration rate); CKD (chronic kidney disease), RRT (renal replacement therapy) ERT (enzyme replacement therapy); CNS (central nervous system).

Vitamin D replacement is recommended in cases of deficiency^47,114^. Some authors recommend paricalcitol for its antiproteinuric effects^43^.

The choice of mode of dialysis is based on individual preference. The outcomes of kidney transplantation in terms of graft and patient survival are similar to transplants performed for other causes. Long-term graft survival might be negatively affected by cardiovascular involvement^115,116^. Recurrence of FD nephropathy after transplantation and in histology has been reported, with no impact on long-term graft survival. Presence of typical lamellar inclusions in transplanted kidneys has been described; they probably originate from invading host macrophages and vascular endothelial cells^115^.

### Kidney therapeutic targets in fd therapy

The kidney targets of specific treatment include controlling proteinuria/albuminuria and stabilizing the GFR or its decline^73,117^, mainly in cases with a baseline GFR below 60 mL/min/1.73m^2 36,47,56^.

The goal is to reduce the annual GFR decline to values less than 3 mL/min/1.73m^2^/year^118^. For patients with rapid kidney involvement progression, decelerating the GFE decline to rates below 5 mL/min/1.73m^2^/year or producing decreases greater than 50% in the rate of progression are significant outcomes^36^. Some patients do not meet the therapeutic target for GFR for presenting with greater tissue damage at the start of therapy^36,75^. Table 8 shows the therapeutic goals for kidney involvement.

## Monitoring adult patients with FD

Care to patients with FD must be based on early assessment and regular functional monitoring of potentially affected organs to check for disease progression, regardless of whether patients are on specific therapy. Therapeutic goals must be individualized and adjusted when needed. Table 9 shows the recommended patient monitoring schedule.

**Table 9:** Organ monitoring schedule for adult patients with FD

Organ/System	Assessment	Frequency
**General**	Medical history and physical examination; assessment of quality of life through scales; performance at school/work; levels of depression and/or anxiety	Every clinical visit.
	*Activity of enzyme* α*-GAL and GLA gene mutation*	If not determined previously.
	Genetic counseling	At the start and as needed.
**Kidney**	GFR	Every year for low risk patients; every six months for moderate risk patients; every three months for high to very high risk patients.
	Albuminuria/proteinuria (24-hour or isolated urine test - protein or albumin-to-creatinine ratio)	Every year for low risk patients; every six months for moderate risk patients; every three months for high to very high risk patients.
	Vitamin D	When clinically indicated.
	Kidney biopsy	When clinically indicated.

Legend: FD (Fabry disease); GFR (glomerular filtration rate).

Note.: When possible, use validated scales for all symptoms and signs related to FD.

Baseline histology analysis, particularly of the kidneys, is used as a parameter to assess disease progression^47,119^.

Monitoring individuals with the late-onset variant is more challenging, since signs and symptoms of FD may appear at the same time as aging-related alterations such as heart and CNS disease. In such cases, cardiac biopsy and T1 mapping of the heart with nuclear magnetic resonance (NMR) imaging with gadolinium enhancement when possible might help differentiate between FD-related injuries from involvements tied to other etiologies^47^.

Asymptomatic females with late-onset variants and normal findings on initial assessment must also be monitored, albeit with longer intervals. The absence of symptoms at diagnosis and during follow-up does not rule out the development of organ complications^47,120^.

Ideal kidney monitoring includes the analysis of the GFR and albuminuria/proteinuria at least annually in patients at low risk of developing CKD, every six months if risk is rated as moderate, and every three months for high-risk patients^47^. In patients on ERT, kidney histology serves as a parameter to assess cases with inadequate response suspected for presenting anti-agalsidase antibodies^34^.

Some patients have shown signs of FD progression despite the administration of specific therapy. Lack of response to treatment may be related to a combination of factors such as late treatment start (presence of irreversible organ damage), incomplete penetration of the infused enzyme in different tissues, lack of proper parameters to detect minor clinical effect, lack of a full understanding of the ERT response mechanism, and the inhibitory effect of anti-IgG antibodies against agalsidase^36,79,121^.

Although screening for anti-IgG antibodies against agalsidase is not considered in current clinical practice, periodic assessment of antibody levels in patients on ERT is recommended, particularly males with classic FD. The higher the levels, the greater the accumulation of GL3 and lyso-GL3, which serves as evidence of inadequate response to therapy^38,79^. However, prospective studies are still needed so that a definitive conclusion is derived about the impact of antibodies and strategies to address these cases are developed^122-124^.

## Genetic counseling recommendations

FD may cause profound emotional and physical impact on patients and their families. In order to better understand the disease, genetic counseling is an essential element in the multidisciplinary effort required in FD care.

Genetic counseling looks into inheritance patterns and includes genetic tests devised to identify affected family members through a pedigree. Females might be just as affected as males and should not be considered solely as carriers of mutation^125^. Genetic counseling must cover psychosocial issues such as anxiety with disease progression, guilt related to the transmission of the disease to the offspring, denial and other emotions such as anger, sadness, hopelessness, and effects on self-esteem and self-identity. Potential economic and social impacts such as disability, unemployment, and life insurance must also be covered^125,126^. Genetic counseling before conception and during prenatal care must be offered to every patient of reproductive age to identify potential inheritance. It is important to advise patients about the potential teratogenic effects of some routine adjuvant therapies^127^.

## Final considerations

The management of FD is still fraught with uncertainty, including the need to more clearly define the role of VUS and the ideal moment to start specific therapy based on the severity of each variant. In cases involving asymptomatic patients, we should assess the possibilities and benefits of developing criteria to individualize drug doses, combine between available therapies, and check whether the standardized evaluation of neutralizing antibodies impacts ERT efficacy. The answers to the questions above require a summation of efforts from everyone involved in FD care.

This consensus document was designed to help manage the expectations of patients and physicians regarding the outcomes of therapy. Our recommendations must be interpreted within the context of evidence. Individual decisions must be made jointly, with the involvement of patients and their families, considering the costs involved - not only the ones of a financial nature, concurrent diseases, and personal preferences.

The Comdora intends to update these recommendations regularly so as to reflect recent literature evidence, real-world data, and appreciate the professional experience of those involved. This consensus document establishes clear criteria for the diagnosis of FD and for when to start or stop specific therapies or adjuvant measures, to thus advise the medical community and standardize clinical practice.
